# Revising the embryonic origin of thyroid C cells in mice and humans

**DOI:** 10.1242/dev.126581

**Published:** 2015-10-15

**Authors:** Ellen Johansson, Louise Andersson, Jessica Örnros, Therese Carlsson, Camilla Ingeson-Carlsson, Shawn Liang, Jakob Dahlberg, Svante Jansson, Luca Parrillo, Pietro Zoppoli, Guillermo O. Barila, Daniel L. Altschuler, Daniela Padula, Heiko Lickert, Henrik Fagman, Mikael Nilsson

**Affiliations:** 1 Sahlgrenska Cancer Center, Institute of Biomedicine, University of Gothenburg, Göteborg SE-40530, Sweden; 2 Department of Surgery, Sahlgrenska University Hospital, Göteborg, SE-41345, Sweden; 3 IRGS, Biogem, Ariano Irpino (AV), 83031, Italy; 4 Institute for Cancer Genetics, Columbia University, 1130 St Nicholas Avenue, New York, NY 10031, USA; 5 Department of Pharmacology and Chemical Biology, School of Medicine, University of Pittsburgh, Pittsburgh, PA 15261, USA; 6 Institute of Diabetes and Regeneration Research, Helmholtz Center Munich, German Research Center for Environmental Health GmgH, Ingolstaedter Landstraße 1, Munich 85764, Germany; 7 Department of Clinical Pathology and Genetics, Sahlgrenska University Hospital, Göteborg, SE-41345, Sweden

**Keywords:** Foxa1, Foxa2, Endoderm, Medullary thyroid cancer, Neural crest, Neuroendocrine

## Abstract

Current understanding infers a neural crest origin of thyroid C cells, the major source of calcitonin in mammals and ancestors to neuroendocrine thyroid tumors. The concept is primarily based on investigations in quail–chick chimeras involving fate mapping of neural crest cells to the ultimobranchial glands that regulate Ca^2+^ homeostasis in birds, reptiles, amphibians and fishes, but whether mammalian C cell development involves a homologous ontogenetic trajectory has not been experimentally verified. With lineage tracing, we now provide direct evidence that Sox17+ anterior endoderm is the only source of differentiated C cells and their progenitors in mice. Like many gut endoderm derivatives, embryonic C cells were found to coexpress pioneer factors forkhead box (Fox) a1 and Foxa2 before neuroendocrine differentiation takes place. In the ultimobranchial body epithelium emerging from pharyngeal pouch endoderm in early organogenesis, differential Foxa1/Foxa2 expression distinguished two spatially separated pools of C cell precursors with different growth properties. A similar expression pattern was recapitulated in medullary thyroid carcinoma cells *in vivo*, consistent with a growth-promoting role of Foxa1. In contrast to embryonic precursor cells, C cell-derived tumor cells invading the stromal compartment downregulated Foxa2, foregoing epithelial-to-mesenchymal transition designated by loss of E-cadherin; both Foxa2 and E-cadherin were re-expressed at metastatic sites. These findings revise mammalian C cell ontogeny, expand the neuroendocrine repertoire of endoderm and redefine the boundaries of neural crest diversification. The data further underpin distinct functions of Foxa1 and Foxa2 in both embryonic and tumor development.

## Abstract (precis)

**Highlighted article:** Mouse thyroid C cell precursors arise in foregut endoderm, and not the neural crest, disproving the current concept of a neural crest origin of thyroid neuroendocrine cells.

## INTRODUCTION

**Fig. 1.**
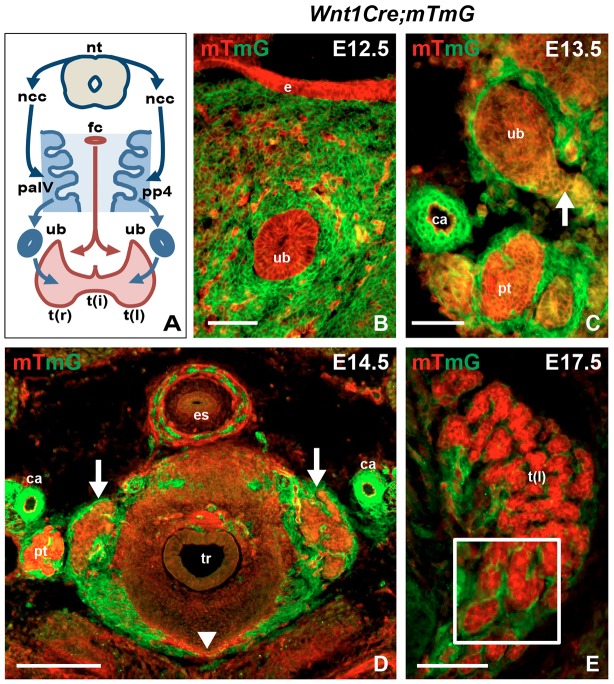
**Thyroid development and contribution of** the **neural crest to the pharyngeal apparatus.** (A) Overview of mammalian thyroid development from a median (red) and two lateral (blue) anlagen and the contribution of neural crest to ectomesenchyme of the pharyngeal apparatus. Arch and pouch numbers refer to mouse embryos. (B-E) Tracing Wnt1+ progeny during thyroid development. *Wnt1-Cre* mice were crossed to *mT/mG* reporter mice. Conversion from red (mT) to green (mG) fluorescence indicates activation of Cre recombinase (Muzumdar et al., 2007). Images are from transverse sections. (B) mT+ ultimobranchial body surrounded by mG+ cells. Scattered mT+ cells are endothelial. (C) mT+ ultimobranchial body merging with the median thyroid primordium (lateral part of the latter indicated with arrow). (D) Orthotopic thyroid after fusion of primordia (arrows indicate the two lobes, arrowhead indicates limited part of the isthmus). (E) Thyroid lobe undergoing follicular organization. Note distribution of Wnt1+ cells limited to the interstitial space in between trabecular parenchyma (inset, magnified part of motif). ca, carotid artery; e, pharyngeal endoderm; es, esophagus; fc, foramen caecum; ncc, neural crest cells; nt, neural tube; pa, pharyngeal arch; pp, pharyngeal pouch; pt, parathyroid; t(i), thyroid isthmus; t(l), left thyroid lobe; t(r), right thyroid lobe; tr, trachea; ub, ultimobranchial body. Scale bars: 200 µm (D); 100 µm (E); 50 µm (B,C).

According to current understanding, C cells of the mammalian thyroid gland develop from a subpopulation of cephalic neural crest cells that broadly populate the pharyngeal (or branchial) arches in all animals of the vertebrate series (Donoghue et al., 2008; Vickaryous and Hall, 2006). The neural crest origin is assumed to feature distinct properties of neuroendocrine thyroid tumors that arise from C cells. For example, in multiple endocrine neoplasia syndromes associated with germline *RET* mutations, medullary thyroid cancer (MTC) coincides with sympathoadrenal tumors, for which the ancestral cells are established neural crest derivatives (Adams and Bronner-Fraser, 2009). However, scientific consensus of a neural crest origin of thyroid C cells relies first and foremost on observations in quail–chick embryo heterografts. These seminal studies enabled tracing of migrating crest cells to diverse locations (Dupin et al., 2006) including the ultimobranchial glands (Le Douarin and Le Lievre, 1970; Polak et al., 1974), which are paired organs that develop from the foregut endoderm of the prospective inferior pharynx. The ultimobranchial glands constitute the major source of calcitonin, a Ca^2+^-regulating hormone, in birds, reptiles and fishes (Copp et al., 1967; Tauber, 1967) but have no close spatial relation to the thyroid in these species. In mammals, the homologous ultimobranchial bodies are transient structures that, soon after delamination from the pharyngeal pouch, coalesce with the embryonic thyroid, thus bringing C cell precursors to it (Pearse and Carvalheira, 1967; Fig. 1A). By analogy, it is generally assumed that thyroid C cells are derived from neural crest. Nonetheless, as no neural crest cells entering the ultimobranchial bodies or the primordial pouch endoderm have ever been unequivocally demonstrated in any mammalian embryo, proof of this concept is yet circumstantial. An alternative germ layer origin of thyroid neuroendocrine cells cannot be excluded.

Using stable reporter constructs, genetic lineage tracing in mice allows imaging of embryonic progenitor cells and their progeny from the onset of the expression of a lineage-specific gene (Blanpain and Simons, 2013; Buckingham and Meilhac, 2011). In this study, we employed a dual approach to elucidate whether embryonic C cell precursors are derived from neural crest or endoderm. Wnt1 expression is restricted to the dorsal neural tube and required for expansion of neural crest cells (Ikeya et al., 1997). Accordingly, tracing *Wnt1*-expressing progeny outside the central nervous system faithfully detects neural crest-derived cells and tissues (Chai et al., 2000; Jiang et al., 2000). By contrast, lineage tracing of foregut endoderm is possible by tracking descendants of progenitors that transiently express Sox17 during formation of definitive endoderm (Engert et al., 2009). Our results provide direct evidence that mouse thyroid C cells develop from pharyngeal endoderm and not neural crest. Moreover, propagation of the C cell lineage involves forkhead box transcription factors (Foxa1 and Foxa2) known to play pivotal roles in organogenesis from foregut endoderm (Kaestner, 2010). These features are recapitulated in human MTC tumors, suggesting involvement in both tumor growth and progression.

## RESULTS

### Wnt1^+^ neural crest progeny does not contribute to the thyroid C cell lineage in mice

**Fig. 2.**
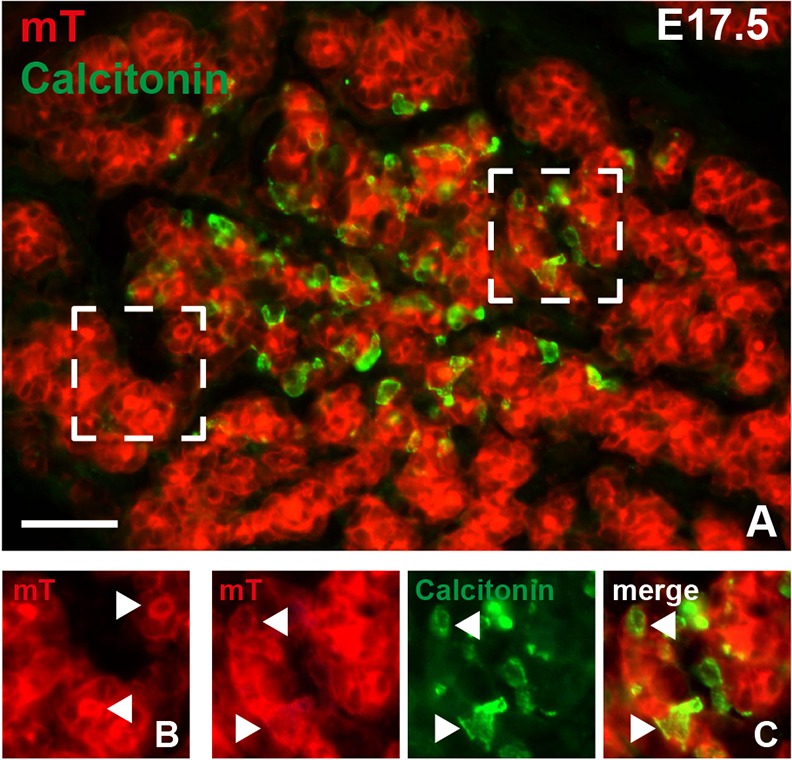
**Localization of embryonic thyroid C cells in *Wnt1-Cre;mT/mG* mouse embryos.** Calcitonin immunoreactivity was detected with streptavidin–fluorescein isothiocyanate. (A) Overview of central part of thyroid lobe. Two areas (dashed boxes) are shown in detail in B (left area) and C (right area). (B) Membranous mT fluorescence in thyroid follicular cells. Note enhanced mT labeling of the apical surface facing the follicle lumen (arrowheads). (C) Thyroid C cells are confined to the Tomato red-positive parenchymal compartment. Note colocalization of mT and calcitonin (arrowheads). Scale bar: 50 µm.

In mice, the ultimobranchial body forms bilaterally from the fourth pharyngeal pouch (Fig. 1A). The pouch is continuous with foregut endoderm and part of an evolutionarily conserved pharyngeal apparatus, from which the thyroid (medially) and the thymus and parathyroid glands (bilaterally) also develop (Graham and Richardson, 2012). The stromal component of the primitive pharynx consists largely of neural crest-derived ectomesenchyme that contributes to further development of the pharyngeal arch and pouch system (Fig. 1A). Based on original findings in quail–chick chimeras (Le Douarin and Le Lievre, 1970), neural crest cells are believed to invade the ultimobranchial bodies before merging with the thyroid primordium. However, whether this indeed occurs in the developing thyroid in mammals has not been experimentally verified. We investigated this by crossing *Wnt1-Cre* mice to a double-fluorescent Cre reporter (*mT/mG*) that allows probe detection with single-cell resolution (Muzumdar et al., 2007). In accordance with results from previous fate-mapping studies using *Wnt1-Cre* and chromogen reporter mice (Chai et al., 2000; Jiang et al., 2000; Jinno et al., 2010; Kameda et al., 2007), mG labeling elicited by the Cre driver was limited to cells of established neural crest origin (Fig. S1A-C). By contrast, pharyngeal endoderm and its derivatives were confined to the red mT compartment (Fig. 1B). Notably, although the ultimobranchial bodies were entirely surrounded by Wnt1+ ectomesenchyme no mG+ cells were encountered inside the ultimobranchial body epithelium (Fig. 1B). Likewise, at the time of fusion with the median thyroid primordium the ultimobranchial bodies excluded cells of the neural crest lineage (Fig. 1C). In subsequent developmental stages, an mG+ prospective capsule enclosed the thyroid lobe, which was also grossly intersected by mG-labeled stromal septations (Fig. 1D). As expected, the follicular cell lineage remained mT+ during folliculogenesis (Fig. 1E). Likewise, differentiated C cells identified by calcitonin immunostaining were exclusively distributed in the mT-labeled parenchymal compartment (Fig. 2). Together, these findings confirm and extend previous notions (Kameda et al., 2007) that Wnt1+ neural crest cells contribute to thyroid connective tissue but are not the source of C cells in the mouse embryonic thyroid.

### Mouse thyroid C cells derive from Sox17^+^ anterior endoderm

**Fig. 3.**
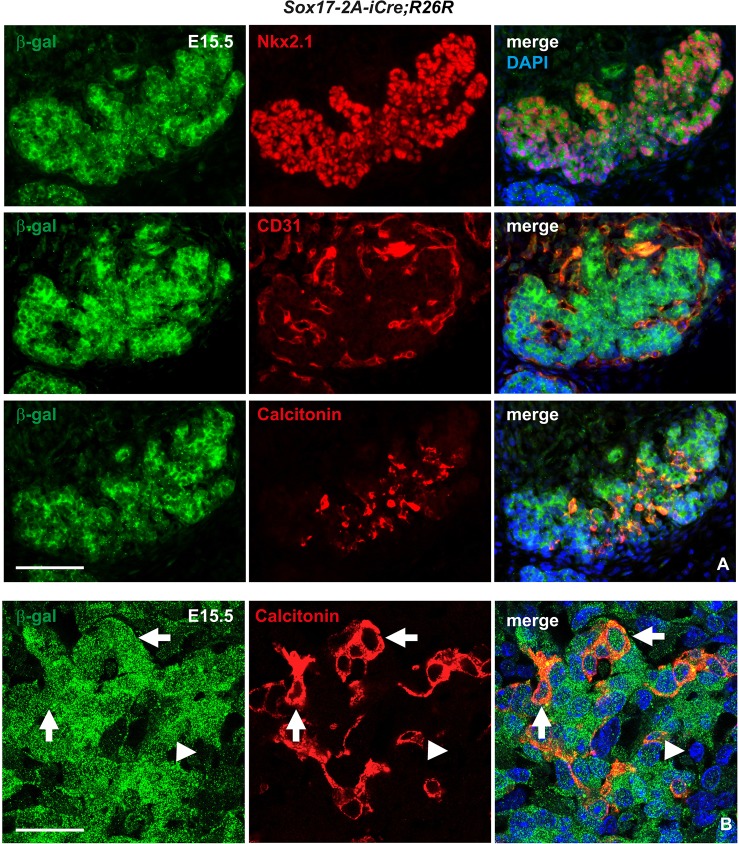
**Tracing Sox17**+ **progeny in embryonic thyroid in *Sox17-2A-iCre;R26R* mice.** Immunofluorescence images of single and merged channels of the indicated markers from serial sections of the same specimen; merged images additionally show 4′,6-diamidino-2-phenylindole (DAPI) nuclear stain. (A) Coexpression of β-galactosidase (β-gal) and Nkx2-1 in follicular cells (upper panel), β-gal and CD31 in endothelial cells (middle panel), and β-gal and calcitonin in C cells (lower panel). (B) Colocalization of β-gal and calcitonin as revealed by confocal laser scan microscopy. Arrows indicate β-gal+ C cells. Arrowheads indicate β-gal– stromal cells. Scale bars: 100 µm (A); 25 µm (B).

Having ruled out the possibility that the origin of C cells is neural crest, we still needed to confirm from which other germ layer these cells arise. Our prime suspect was endoderm and, specifically, the pharyngeal pouch domain that develops into the ultimobranchial bodies. Definitive endoderm is established under the influence of the SRY (sex-determining region Y)-box 17 (Sox17) transcription factor (Engert et al., 2009). As its endodermal expression disappears before organ-specific domains are discernible and because Sox17 is excluded from mesenchyme apart from mesoderm-derived microvessels (Engert et al., 2009), lineage tracing of Sox17+ descendants should be decisive to determine whether thyroid C cells derive from endoderm or not. Analysis of *Sox17-2A-iCre;R26R* mice employing a β-gal reporter showed, as expected, that the entire pharyngeal endoderm was X-gal positive (Fig. S2A). Notably, this included the thyroid bud and ultimobranchial bodies, in contrast to surrounding β-gal–mesenchyme (Fig. S2B). *Sox17-Cre*-mediated labeling was thus mutually exclusive to that of *Wnt1-Cre*. At later developmental stages, β-gal immunostaining of these mice showed ubiquitous coexpression in Nkx2-1+ parenchymal cells and CD31 (Pecam1 – Mouse Genome Informatics)+ endothelial cells present in the embryonic thyroid (Fig. 3A). This also demonstrated that calcitonin-expressing cells appearing at embryonic day (E) 15 onwards were confined to β-gal+ thyroid parenchyma (Fig. 3A). To rule out any possible contribution of superimposed immunoreactivity from adjacent follicles, β-gal expression in C cells was confirmed by confocal imaging (Fig. 3B). These lineage-tracing results conclusively demonstrated that mouse thyroid C cells are derived from progenitors originating in Sox17+ anterior endoderm.

### Foxa1 and Foxa2 are differentially expressed in C cell precursors

**Fig. 4.**
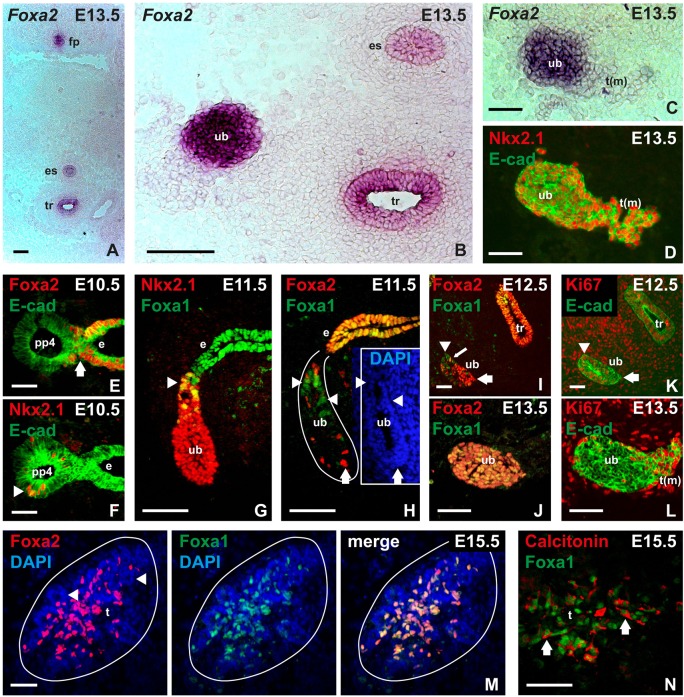
**Expression of forkhead box transcription factors Foxa1 and Foxa2 in mouse ultimobranchial bodies, C cell precursors and embryonic C cells.** Analysis of wild-type embryos with *in situ* hybridization (A-C) and immunofluorescence (D-N), respectively. Endoderm and derivatives thereof were identified with E-cadherin (E-cad) as indicated. (A) Overview of *Foxa2* expression in midline structures. (B) *Foxa2* expression in the ultimobranchial body (image from a more posterior section to that shown in A). (C,D) *Foxa2* at fusion of ultimobranchial body with midline thyroid as identified by Nkx2-1 expression (data from serial sections of the same specimen). (E) Loss of Foxa2 expression in the prospective ultimobranchial body; arrow indicates transition between fourth pharyngeal pouch and endoderm proper. (F) Nkx2-1+ cells (arrowhead) exclusively present in the fourth pouch of lateral pharyngeal endoderm. (G) Differential expression of Foxa1 (arrowhead) in the emerging Nkx2-1+ ultimobranchial body. (H-J) Distribution of Foxa1+ (arrowheads) and Foxa2+ (arrows) cells in the developing ultimobranchial body from the time of delamination (H) to fusion with midline thyroid (J). Inset (in H) outlines the ultimobranchial body epithelium co-stained with DAPI. Small arrow (in I) indicates a single Foxa1+/Foxa2+ cell. (K,L) Distribution of Ki67+ ultimobranchial body cells before (K) and at fusion with midline thyroid (L). Arrow and arrowhead in K correspond to the same labels in I for comparison with Foxa1/Foxa2 expression pattern. (M) Coexpression of Foxa1 and Foxa2 in C cell precursors dispersed in the thyroid lobe after fusion of primordia. Arrowheads indicate follicular parenchyma with DAPI staining only. (N) Coexpression of Foxa1 and calcitonin in embryonic thyroid C cells. Note separate staining of markers (arrows) confined to nucleus and cytoplasm, respectively. e, pharyngeal endoderm; es, esophagus; fp, floor plate; pp, pharyngeal pouch; t, embryonic thyroid; t(m), midline thyroid primordium; tr, trachea; ub, ultimobranchial body. Scale bars: 100 µm (C,D,G,H); 50 µm (A,B,E,F,I-L); 25 µm (M,N).

Endodermal organs regularly express the forkhead box transcription factor Foxa2 and often also its closely related paralog Foxa1 (Kaestner, 2010). Foxa2 is essential for the formation and patterning of definitive endoderm foregoing fate determination of organ-specific progenitors. Accordingly, lineage tracing of Foxa2+ progeny labeled the entire pharyngeal endoderm including the thyroid bud and the pharyngeal pouch, from which the ultimobranchial bodies emerge (Fig. S2C,D). *In situ* hybridization showed that the mature ultimobranchial body expressed *Foxa2* in a similar manner to the prospective respiratory and esophageal epithelia and floor plate (Fig. 4A,B). Notably, *Foxa2* transcripts were not detected in the thyroid primordium, confirming previous findings that Foxa2 is downregulated in the follicular lineage already at the bud stage (Fagman et al., 2011). In fact, *Foxa2* expression defined a distinct border between midline thyroid and ultimobranchial body cells as these tissues approached and coalesced at E13.5 (Fig. 4C,D). Importantly, this enabled a labeling strategy to distinguish lineage progenies from the respective anlagen and potentially identify embryonic C cell precursors before they started to differentiate and express calcitonin in late thyroid development.

In the mouse, the ultimobranchial bodies emerge from the fourth pharyngeal pouches at E10. At this stage, when only a few ultimobranchial cells expressed Nkx2-1, it was evident that Foxa2 was specifically downregulated in the entire pouch domain of the endoderm (Fig. 4E,F). Loss of Foxa2 also characterized the third pharyngeal pouch, from which thymus and parathyroid glands derive (data not shown). As diminished Foxa2 expression in these transient pharyngeal structures is shared by the thyroid bud but not lung and liver primordia (Fagman et al., 2011; Lee et al., 2005), this feature might reflect a common mechanism of disbudding organs that entirely lose contact with the prospective gut tube in early organogenesis. Unlike endoderm in general, the pouch endoderm was also negative for Foxa1 (data not shown).

Upon delamination, nearly all ultimobranchial cells were Nkx2-1 positive and started to re-express both Foxa1 and Foxa2 (Fig. 4G,H). Notably, this appeared in a distinct pattern along the axis of the ultimobranchial body, i.e. Foxa1+ cells were mainly located in the proximal part still connected to the pharyngeal pouch, whereas Foxa2+ cells were scattered in the distal Foxa2– tip (Fig. 4H). Separation of Foxa1+ and Foxa2+ cells was also evident after the ultimobranchial body lost contact with the endoderm. Interestingly, this reflected different growth properties within the epithelium in that Ki67 labeling coincided with Foxa1 in the rear and Ki67– cells predominated in the Foxa2+ ventral domain (Fig. 4I,K). One day later, when the ultimobranchial body started to merge with the midline thyroid primordium, most if not all cells coexpressed Foxa1 and Foxa2 (Fig. 4J). At the same time, the entire epithelium became Ki67–, in contrast to the adjacent midline thyroid and surrounding mesenchyme (Fig. 4L). Together, this suggests that ultimobranchial cells in early development consist of two populations preferentially expressing Foxa1 or Foxa2, correlating with high and low growth rate, respectively, and that this separation is lost in favor of a common Foxa1+Foxa2+ phenotype that ceases to proliferate before C cell precursors enter the embryonic thyroid.

### Foxa1 and Foxa2 are coexpressed in thyroid C cells

The compound embryonic thyroid gland is established as the ultimobranchial body disintegrates and its cells spread bilaterally into the respective lobes (Fagman et al., 2006). Notably, Foxa2 distinguished this cell population from thyroglobulin+ follicular cells as the two lineages gradually mixed (Fig. S3A,B). Foxa2+ cells migrating peripherally also colocalized with the distribution of differentiated C cells, indicating that they were probably identical (Fig. S3B,C). This notion was confirmed by double immunostaining in E15.5 embryos; dispersed cells of ultimobranchial origin coexpressed Foxa1 and Foxa2 (Fig. 4M) and calcitonin (Fig. 4N). Adult thyroid C cells also expressed Foxa2 (Fig. S3D,E).

### Foxa1 and Foxa2 are differentially expressed in human medullary thyroid carcinoma

As Foxa1 and Foxa2 distinguished C cell precursors with different growth characteristics but were coexpressed in differentiated C cells, we investigated whether neoplastic C cells might recapitulate any of these features. MTC accounts for 5-8% of all thyroid cancers and occurs either sporadically or as a familial trait (Pacini et al., 2010). Hereditary MTC, whether isolated or part of multiple endocrine neoplasia, is caused by activating *RET* mutations; one-third of sporadic cases has the same pathogenesis. Indolent tumor growth and diffuse symptomatology delay diagnosis, leading to MTC often being uncovered at an advanced stage with distant metastases (Pacini et al., 2010). Notably, apart from constitutive activation of the mitogen-activated protein kinase signaling pathway (driven by mutant RET), the mechanisms of MTC tumor development and progression are largely unknown.

**Fig. 5.**
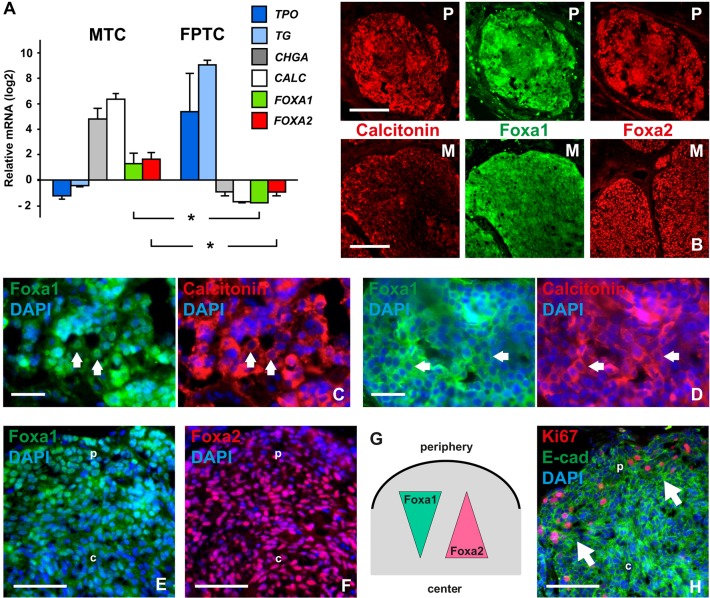
**Expression of Foxa1 and Foxa2 in neuroendocrine thyroid cancer.** (A) Comparison of microarray data sets from human medullary thyroid carcinoma (MTC; *n*=9) and follicular variant of papillary thyroid cancer (FPTC; *n*=7). *FOXA1*/*FOXA2* expression levels relative to mRNAs of biomarkers for the respective tumor type (CALC, calcitonin; CHGA, chromogranin A; TG, thyroglobulin; TPO, thyroid peroxidase) are shown as the difference from the mean of all genes in each data set. Asterisks indicate *P*<0.001. Error bars represent s.d. (B-F) Immunofluorescence staining of Foxa1 and Foxa2 in tumor tissues from an MTC patient. Sections were co-stained for calcitonin to distinguish tumor from stromal cells. (B) Nodules from primary tumor (P) and lymph node metastasis (M) at low magnification. (C,D) Detailed views of tumor tissues with different cell densities. Arrows indicate accumulation of Foxa1 predominantly in nuclei (in C) or cytoplasm (in D) associated with low and high cellularity, respectively. (E) Nuclear accumulation of Foxa1 differs between peripheral (p) and central parts (c) of tumor nodule. (F) Expression levels of Foxa2 in peripheral (p) versus central parts (c) of tumor nodule. (G) Distinct expression pattern of Foxa1 and Foxa2 in MTC related to tumor tissue organization (depicted from data shown in E,F). Increase/decrease refers to nuclear accumulation. (H) Distribution of proliferating cells in tumor nodule. Arrows indicate clusters of Ki67+ cells in the peripheral zone (p) facing the tumor stroma. Ki67+ cells are not present in the nodule center (c). Scale bars: 100 µm (B); 25 µm (C,D); 50 µm (E,F,H).

We first evaluated publicly deposited microarray data sets obtained from human thyroid malignancies. This showed that both *FOXA1* and *FOXA2* transcripts were enriched in MTC compared with thyroid cancer of follicular cell origin (Fig. 5A). High expression levels of *FOXA1* and *FOXA2* were also evident in a *RET*-mutant MTC cell line (TT) but not in established lines from other types of thyroid carcinoma (Fig. S4A). This confirmed lineage specificity of Foxa1 and Foxa2 as being exclusively expressed in C cells and further indicated that human and mouse thyroid do not differ in this respect.

Next, tumor tissues sampled directly at surgery of an MTC patient with disseminated disease were processed in an identical manner to mouse embryos for evaluation by immunofluorescence. This showed generally strong immunoreactivity for Foxa1 and Foxa2 in both primary tumor nodules and lymph node metastases (Fig. 5B). Notably, Foxa1 was predominantly nuclear in loosely coherent cells (Fig. 5C) but mostly cytoplasmic in more crowded nests of tumor cells (Fig. 5D). Nuclear localization of Foxa1 typically distinguished the peripheral zone from central portions of tumor nodules (Fig. 5E,G). By contrast, although most cells showed nuclear staining of Foxa2 the expression level was weaker or occasionally lost towards the tumor periphery facing the stromal compartment (Fig. 5F,G).

### Foxa1 promotes proliferation of malignant thyroid C cells

Given the putative growth-regulatory role of Foxa1 in mouse C cell precursor subsets (Fig. 4), its differential expression in MTC predicted that cancer cell proliferation might be patterned in a similar manner depending on the tumor context. In support of this hypothesis, we found that Ki67+ cells accumulated in the peripheral zone of tumor tissues (Fig. 5H). A functional role was investigated further in TT cells subjected to knockdown of *FOXA1* and *FOXA2*, respectively (Fig. S4B,C). This significantly reduced DNA synthesis after silencing of *FOXA1* only (Fig. S4D). Collectively, these observations indicate that nuclear Foxa1 promotes growth of neoplastic C cells.

### Foxa2 is transiently downregulated in invasive medullary thyroid cancer cells that undergo epithelial-to-mesenchymal transition

**Fig. 6.**
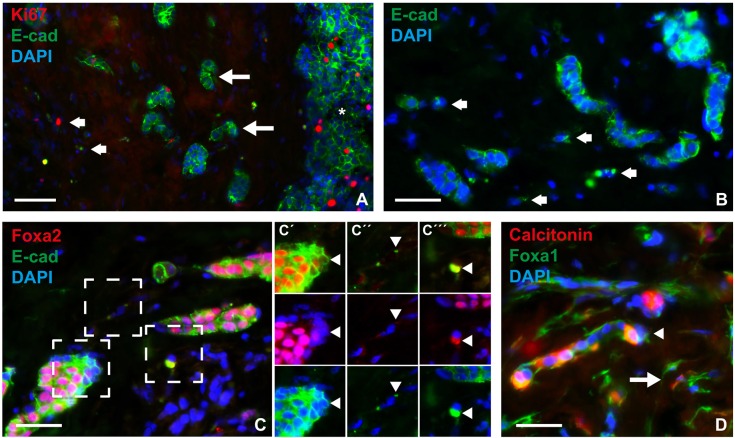
**Altered Foxa2 expression in invasive MTC tumor cells.** Sections were co-stained for E-cadherin (E-cad) to evaluate concurrent epithelial-to-mesenchymal transition. (A) Overview of tumor invasive zone. Large arrows indicate clusters of infiltrating tumor cells close to a large tumor nodule (asterisk); small arrows indicate distanced single tumor cells. Note that Ki67+ cells are not enriched in the tumor stroma compared with solid tumor nodule. (B) Redistribution of E-cadherin from the surface of single tumor cells (arrows). (C) Coordinated loss of Foxa2 in invasive tumor cells. Three areas (dashed boxes) of motif are shown in C′-C‴ with alternating channels for improved resolution: Foxa2 and E-cad (upper panel); Foxa2 and DAPI (middle panel); and E-cad and DAPI (lower panel). Arrowheads indicate different cell phenotypes further commented on in the Results. (D) Preserved expression of Foxa1 confined to the cytoplasm in infiltrating tumor cells. Note variable expression of calcitonin in both single tumor cells (arrow) and small aggregates (arrowhead). Scale bars: 50 µm (A); 25 µm (B-D).

It is already known that Foxa2 downregulation is required to elicit epithelial-to-mesenchymal transition (EMT) in various cell lines established from malignant tumors originating in endoderm-derived organs (Song et al., 2010; Tang et al., 2011). Loss of Foxa2, as observed in MTC cells in close contact with the tumor stroma, suggested that a similar mechanism might facilitate tumor spreading *in vivo*. We investigated this possibility by monitoring Foxa2 in infiltrating tumor cells that displayed signs of EMT. E-cadherin (Cadherin 1), the loss of which is a hallmark of EMT, was strongly expressed in the vast majority of MTC cells in both primary tumor (Fig. 5H) and metastatic sites (data not shown). This also accounted for smaller solid nests and cords of tumor present in the stromal compartment (Fig. 6A,B). However, scattered single cancer cells displayed residual amounts of E-cadherin that mostly had a non-membranous distribution. Notably, loss of Foxa2 expression was evident in edge cells of E-cadherin+ tumor nests (Fig. 6C,C′). Moreover, single tumor cells with only patchy remnants of E-cadherin were Foxa2– (Fig. 6C,C″) or showed strong E-cadherin staining that colocalized with Foxa2 in the cytoplasm (Fig. 6C,C‴). There were few Ki67+ cells outside larger tumor nodules, suggesting that emigrating tumor cells ceased to proliferate. In support of this notion, most if not all infiltrating tumor cells showed cytoplasmic Foxa1 staining, which also revealed spindle-type cell shapes conspicuous in EMT (Fig. 6D). Calcitonin expression was variably lost in invasive tumor cells (Fig. 6D). Taken together, these findings indicate that the expression level and nucleocytoplasmic distribution of Foxa2, but not that of Foxa1, is dynamically altered in MTC cells during local spreading along with signs of dedifferentiation and EMT. In this process, downregulation of Foxa2 foregoes that of E-cadherin. Cytoplasmic colocalization of Foxa2 and E-cadherin might reflect neosynthesis as new tumor cell clusters form.

## DISCUSSION

Based on lineage-tracing experiments in mouse embryos, we provide direct evidence that thyroid C cell precursors arise in the endoderm. Progenitors residing in Sox17+ anterior endoderm are committed to a C cell fate hallmarked by Foxa1 and Foxa2 coexpression as glandular development from the pharyngeal part of the foregut takes place. The ultimobranchial bodies emerging from the posterior-most pair of the pharyngeal pouches eventually consist of a homogeneous Foxa1+Foxa2+ epithelial cell population that, after fusion of organ primordia, disseminate in the embryonic thyroid parenchyma and differentiate into C cells. These findings disprove the current concept of a neural crest origin of thyroid C cells.

The possibility that C cells in mammals and other vertebrates, in which the ultimobranchial gland but not the thyroid produces calcitonin, evolved by different mechanisms is debatable. Convergent genesis of identical phenotypes from distinct developmental origins might indeed take place, as recently reported and discussed (Graham, 2010; Jinno et al., 2010). However, as the ultimobranchial bodies are likely to be endodermally derived in all vertebrate classes it is necessary to consider the possibility that the quail–chick experiments, on which the neural crest story of C cells was originally based, might not be understood fully in every detail and the results might be interpreted incorrectly. From an evolutionary viewpoint, it seems more likely that ultimobranchial bodies in all species share the basic developmental program and that their incorporation in thyroid, unique to the mammalian gland and guided by Nkx2-1 (Kusakabe et al., 2006), is an adaptation of the same developmental trait.

Our findings conceivably explain a number of incidental observations that have previously raised doubts about the neural crest origin of thyroid C cells, highlighted in a recent review (Adams and Bronner-Fraser, 2009). In short, this comprises the following aspects. Nkx2-1, implicated in organ development from foregut endoderm (i.e. thyroid and lung), is expressed in differentiated C cells (Mansouri et al., 1998) and required for their survival embryonically (Kusakabe et al., 2006), but Nkx2-1 has no identified role in established neural crest lineages. Lingual thyroids resulting from impaired delamination of the thyroid bud from the pharyngeal floor in early organogenesis contain C cells (Vandernoot et al., 2012), indicating that neuroendocrine development might occur in an ectopic thyroid gland. C cells are also encountered in the orthotopic thyroid in patients with DiGeorge syndrome (Pueblitz et al., 1993), in which neural crest colonization of the pharyngeal arches, and hence pharyngeal pouch organogenesis, is impaired (Liao et al., 2004; Vitelli et al., 2002). In both cases, it is unlikely that ultimobranchial bodies contributed cells to the thyroid, because of either an anatomically distant origin of development or agenesis. The possibility of a mutual lineage origin in endoderm of parafollicular C cells and follicular epithelial cells has also been suggested based on a rare tumor phenotype of mixed medullary and follicular thyroid cancer (Matias-Guiu, 1999). Notably, both normal C cells and MTC tumor cells are able to form follicles *in vivo* (Harach and Williams, 1983), suggesting common traits of epithelial organization.

**Fig. 7.**
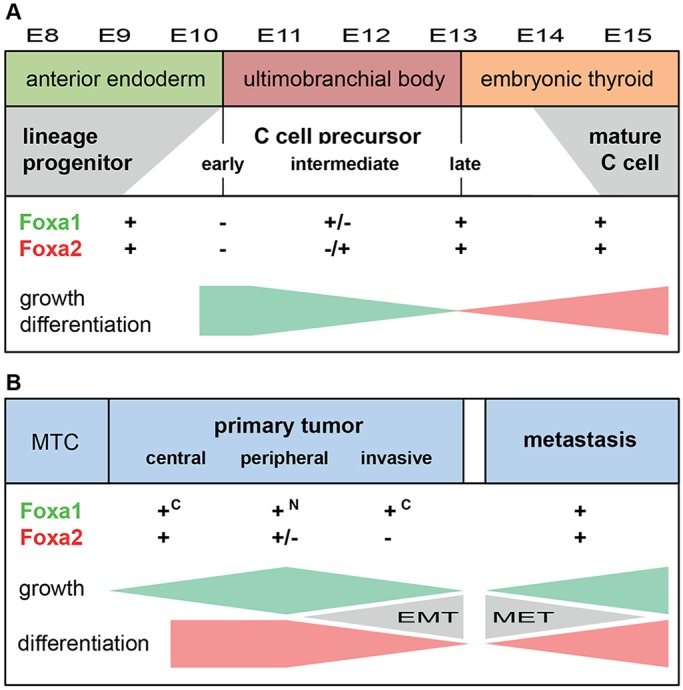
**Lineage development of mouse thyroid C cells and mechanisms of tumor progression in human medullary thyroid cancer proposed from findings in the present study.** Altered expression of Foxa1 and Foxa2 is correlated with growth (represented by green gradients) and differentiation (represented by pink gradients) in a spatiotemporally distinct pattern that shows both similarities and differences between embryonic C cell precursors (A) and neoplastic C cells (B). See Discussion for further comments. c, cytoplasmic localization; EMT, epithelial-to-mesenchymal transition; MET, mesenchymal epithelial transition; MTC, medullary thyroid carcinoma; N, nuclear localization.

In mid-gestation, different domains of the emerging ultimobranchial body epithelium showed differential expression of Foxa1 and Foxa2 that were correlated with Ki67 staining and also previous data from bromodeoxyuridine labeling (Fagman et al., 2006). This suggests that Foxa1 rather than Foxa2 is involved in the proliferation of endoderm progenitors committed to a C cell fate. Indeed, recent genome-wide binding location analysis has revealed that Foxa1 and Foxa2, although sharing many features and being required in concert, e.g. for liver specification (Lee et al., 2005), exert divergent functions that are probably determined evolutionarily following gene duplication (Bochkis et al., 2012). Based on distinct clustering of unique DNA-binding targets, this hypothesis infers Foxa1 as the ancestral gene implicated in cell cycle control, whereas Foxa2 acquired novel properties employed for coordination of the transcriptional network that regulates functional differentiation (Bochkis et al., 2012). It is noteworthy that the calcitonin/calcitonin gene-related peptide enhancer contains a functional Foxa2 binding site (Viney et al., 2004). Nonetheless, we found that Foxa2 is induced in C cell precursors long before terminal differentiation, suggesting an additional morphogenetic role. Given that Foxa2 promotes the epithelial phenotype both in embryonic development (Burtscher and Lickert, 2009) and in adult cells (Tang et al., 2011), it is possible that re-expression of Foxa2 in the developing ultimobranchial body is required for induction of a molecular program that keeps cell cohesiveness and epithelial integrity. From this, we hypothesize that Foxa1 stimulates proliferation of C cell precursors and that Foxa2 promotes epithelial differentiation by preventing premature dissolution of the ultimobranchial body epithelium as it delaminates from pharyngeal endoderm (Fig. 7A). Given that Foxa2 expression continues as C cells spread into the embryonic thyroid, it is likely that other yet unknown mechanisms allow a timely dissemination in late organogenesis.

Distinct roles of Foxa1 and Foxa2 are also consistent with our findings in C cell-derived thyroid cancer, suggesting that part of the developmental program is recapitulated in a tumor context (Fig. 7B). Although both factors were ubiquitously expressed in most MTC cells, a differential expression pattern was evident in two locations of the tumor: the peripheral zone with enrichment of proliferating Ki67+ cells and the invasive front consisting mostly of Ki67– cells. Tumor cell proliferation was correlated with nuclear accumulation of Foxa1, and a direct role of Foxa1 in MTC growth regulation was also indicated in knockdown experiments *in vitro*. By contrast, loss of Foxa2 expression characterized invasive tumor cells that showed definitive signs of EMT. These findings strongly suggest that Foxa1 and Foxa2 exert different and partly opposing effects in MTC tumor growth and progression.

Foxa2 has been reported to suppress EMT and inhibit motility and invasiveness of cancer cell lines derived from tumors of endodermal origin (Song et al., 2010; Tang et al., 2011). Preservation of the epithelial phenotype depends on a direct inhibitory action on Slug (Snai2), which if unleashed drives EMT by repressing the expression of E-cadherin. However, whether tumor progression *in vivo* involves a similar mechanism governed by downregulation of Foxa2 was previously unknown. The present data suggest that loss of Foxa2 is a transient phenomenon confined to a subset of invasive cells and that Foxa2 is re-expressed in metastatic locations accompanying regain of the epithelial phenotype. Transitional states with cytoplasmic localization of both Foxa2 and E-cadherin suggestive of neosynthesis in single MTC cells further support the idea that EMT is a highly dynamic and reversible process, possibly influenced by the immediate tumor cell microenvironment.

It was recently observed in breast cancer patients that circulating tumor cells exhibiting a predominantly mesenchymal phenotype are probably derived from rare primary tumor cells with bi-phenotypic (both epithelial and mesenchymal) traits (Yu et al., 2013). According to this model, partial EMT is required for tumor cells to enter the circulation, but epithelial features reappear after extravasation at secondary sites (Thiery and Lim, 2013). In MTC, based on the present findings, it is conceivable that infiltrating tumor cells with diminished Foxa2 and E-cadherin expression are more prone to metastasize than clusters of Foxa2+ cells that are constrained by E-cadherin-based adhesion. It is noteworthy that C cell precursors migrating into the embryonic thyroid do so by individual rather than collective migration that is reminiscent of tumor cells invading a stromal compartment. However, as embryonic C cells sustainably express both Foxa2 and E-cadherin (present study; Kameda et al., 2007) it is likely that a drive to undergo EMT comprising loss of Foxa2 and E-cadherin specifically characterizes the invasive tumor phenotype (Fig. 7B).

In summary, we show here that neuroendocrine cells of the mammalian thyroid gland are derived from foregut endoderm, indicating that the current view referred to in major medical textbooks (that those thyroid C cells are neuroectodermal and of neural crest origin) is fallacious. This discovery further argues that the tumor entity of MTC should be reclassified to the family of neuroendocrine tumors with endodermal ancestry. Consistent with a key role in neuroendocrine tumor development (Chiaverotti et al., 2008; Khoor et al., 2004; Qi et al., 2010), MTC cells differentially express transcription factors Foxa1 and Foxa2, correlating with tumor growth and invasion. This partly recapitulates the morphogenetic pattern of Foxa1 and Foxa2 as observed during embryonic development of C cell precursors.

## MATERIALS AND METHODS

### Animals and lineage tracing

Animal handling and experiments were approved by the institutional ethic committees at the respective universities in Gothenburg, Naples, Munich and Pittsburgh according to regulations and guidelines of the European Union and the USA. Wild-type (C57BL/6 and CD1) mice were purchased from Charles River Laboratories. *Wnt1-Cre* (JAX009107) and double fluorescent *mT/mG* reporter [JAX 007676; B6.129(Cg)-Gt(ROSA)26Sortm4(ACTB-tdTomato,-EGFP)Luo/J; homozygous] mouse lines were from Jackson Laboratories. *Sox17-2A-iCre* and *Foxa2-2A-iCre* lines were generated and used as previously described (Engert et al., 2009; Horn et al., 2012). *Wnt1-Cre* was crossed to *mT/mG*. *Sox17-2A-iCre* and *Foxa2-2A-iCre* were crossed to the *R26R* Cre reporter (C57BL/6 background). Embryos were sampled at the indicated times. Genotyping by PCR analysis on tail-tip genomic DNA was performed as follows: for *Wnt-Cre*, according to http://jaxmice.jax.org/protocolsdb/f?p=116:2:0::NO:2:P2_MASTER_PROTOCOL_ID,P2_JRS_CODE:4789,009107; for *Sox17*, using a forward primer in the coding region of exon 5 (EP400: 5′-GTGTATAAGCCCGAGATGG-3′) combined with a reverse primer in the 3′ end of exon 5 (EP401: 5′-CTCAACTGTTCAAGTGGCAG-3′) to give products of 470 and 288 bp for the targeted and wild-type alleles, respectively; for *Foxa2*, in exon 3, forward (EP397: 5′-CTACTACCAAGGAGTGTACTCC-3′) and reverse (EP398: 5′-CTGTGGCCCATCTATTTAGGG-3′) primers yielding targeted and wild-type products of 457 and 207 bp, respectively. Genotyping of R26R Cre reporter strain (background C57BL/6) was performed by PCR as described (Soriano, 1999).

### Tumor samples and expression profiling

Raw data from publicly deposited human gene expression profiling experiments available in .CEL format were analyzed for the expression of *Foxa1* and *Foxa2* in comparison to that of the following established thyroid cancer biomarkers: thyroglobulin, thyroperoxidase, calcitonin and chromogranin A. The first data set, consisting of nine MTC samples obtained from multiple endocrine neoplasia type 2B (MEN2B) patients, were hybridized on Affymetrix GeneChip HG-U95Av2 arrays (Ippolito et al., 2005; Jain et al., 2004), and the second data set of seven papillary thyroid cancer (follicular variant) tumors obtained from the International Genomics Consortium (expO; http://www.intgen.org) were hybridized on Affymetrix GeneChip Human Genome U133 Plus 2.0 arrays (GSE1209). The data thus obtained from different platforms were separately analyzed using R/bioconductor (http://www.R-project.org/, http://www.bioconductor.org/), in which both data sets were normalized using robust multi-array average (RMA; Irizarry et al., 2003). Expression values were calculated as differences from each data set mean to allow comparison of transcription levels. Statistical analysis used the *t*-test.

Tumor biopsies dispensable for diagnosis were obtained at debulking surgery of a 54-year-old male MTC patient with disseminated disease, following informed consent. Tissue samples kept in Earl's minimal essential medium on ice were trimmed to a suitable size, fixed in 4% paraformaldehyde and further processed for cryosectioning and immunostaining following the protocol used for embryos (see next subsection).

### β-galactosidase staining and histology

For whole-mount β-gal staining, after fixation in 1% formaldehyde and 0.2% glutaraldehyde in a PBS (pH 7.3) solution containing 0.02% NP-40, 5 mM EGTA (pH 8.0) and 2 mM MgCl_2_ for 60 min at room temperature, embryos were incubated with 1 mg/ml X-gal dissolved in 0.02% NP-40, 2 mM MgCl_2_, 5 mM K_3_[Fe(CN)_6_], 5 mM K_4_[Fe(CN)_6_]·6H_2_O and 0.01% sodium deoxycholate overnight at 37°C under gentle shaking. Washing in PBS (pH 7.3) containing 0.02% NP-40 followed each step. Embryos were post-fixed and stored in 4% paraformaldehyde at 4°C until dehydrated through an ethanol series and embedded in paraffin. Sections (10 µm thick) were counterstained with Nuclear Fast Red and viewed and photographed in a Zeiss Axioscope 2 Plus microscope. Embryos older than E10.5 showed inhomogeneous β-gal staining in deeper tissues, including thyroid, and were not analyzed further.

### Immunofluorescence

Intact (E13 and younger) or dissected embryos (>E13, decapitated and cross-sectioned below the heart) were fixed in 4% paraformaldehyde overnight at 4°C and, after cryoprotection in 30% sucrose in PBS, embedded in TissueTek OCT and frozen at −80°C. Cryosections (10 µm thick) collected on glass slides were subjected to pairwise double immunolabeling as indicated with the following antibodies: rabbit anti-Nkx2-1 (Biopat, Italy; PA0100, 1:1000), rabbit anti-calcitonin (DAKO; A0576, 1:500), guinea pig anti-Foxa1 (kindly provided by Jeffrey Whitsett, Cincinnati Children's Hospital, OH, USA; 1:2000; applied for mouse tissue only), mouse anti-Foxa1 (Seven Hills Bioreagents; WMAB-2F83, 1:1000; applied for human specimens only), rabbit anti-Foxa2 (Seven Hills; WRAB-FOXA2, 1:2000), rat anti E-cadherin (ECCD2; Calbiochem; 205604, 1:1000), rat anti-CD31/Pecam-1 (BD Biosciences; 557355, 1:250), chicken anti-β-gal (Abcam; ab9361, 1:2000) and rabbit anti-Ki67 (Abcam; ab16667, 1:100). Secondary antibodies for detection were rhodamine red-X-conjugated anti-rabbit IgG (Jackson ImmunoResearch), biotin-conjugated anti-rat or anti-guinea pig IgGs (Jackson ImmunoResearch) followed by streptavidin–fluorescein isothiocyanate (DAKO) and, for confocal imaging, Alexa Fluor 555 donkey anti-rabbit IgG (Invitrogen) and Alexa Fluor 488 donkey anti-chicken IgG (Jackson ImmunoResearch). Images were captured in a Zeiss Axioscope 2 Plus fluorescence microscope or a Zeiss LSM 700 laser scanning microscope.

### *In situ* hybridization

*In situ* hybridization of *Foxa2* on quick-frozen CD1 mouse embryos was performed essentially as previously reported (Fagman et al., 2011). A plasmid containing the complete mouse Foxa2 cDNA (kindly provided by Klaus Kaestner, Translational Research Center, Perelman School of Medicine, University of Pennsylvania, PA, USA) was used as template for riboprobe synthesis following a protocol from the GenePaint consortium (Fagman et al., 2011). Hybridization followed the protocol described by Little et al. (2007). Images captured by a Zeiss Axioplan2 microscope equipped with an Axiocam digital camera were processed using Axion Vision software and the ImageJ software. No signal was detected with the sense riboprobe (data not shown).

### PCR analysis

Established cell lines from thyroid cancer subtypes (TT from medullary, TPC1 and BCPAP from papillary, and C643 and SW1736 from anaplastic tumors, respectively) were sampled for PCR analysis of *FOXA1* and *FOXA2*. Total RNA was isolated with RNeasy Micro Kit (Qiagen). cDNA was synthesized using the GeneAmp RNA PCR Kit (Applied Biosystems) followed by amplification of 1 µl of cDNA per sample for further analysis in an ABI Prism 7000 Sequence Detection System (Applied Biosystems). Primer sequences and PCR product size were as follows: *FOXA1*, 5′-GAAGATGGAAGGGCATGAAACCA-3′ and 5′-TGGCATAGGACATGTTGAAGGACG-3′ (194 bp); and *FOXA2*, 5′-CTACGCCAACATGAACTCCA-3′ and 5′-GAGGTCCATGATCCACTGGT-3′ (199 bp). *ACTB* mRNA was used as an internal control. TT cells were also subjected to quantitative real-time PCR (qPCR) analysis of *FOXA1* (Hs00270129_m1) and *FOXA2* (Hs00232764_m1) using Taqman Gene Expression Assays and the AB 7500 Fast Real-Time PCR System (Applied Biosystems). The relative expression levels of *FOXA1* and *FOXA2* were calculated with SDS Software v1.4 (Applied Biosystems) using *GAPDH* (Hs99999905_m1; Applied Biosystems) as the reference gene on quadruplicate samples.

### RNA interference

TT cells were incubated with Silencer^®^ Select (Ambion) predesigned siRNA products against *FOXA1* (s6687, s6688 and s6689) and *FOXA2* (s6690, s6691 and s6692). Similar results were obtained for all *FOXA1* siRNAs; data shown in the present study used s6688 [ACAUGACCAUGAACACCAUtt (sense); AUGGUGUUCAUGGUCAUGUag (antisense)]. Efficient suppression of *FOXA2* was observed only with s6692 [UGAACGGCAUGAACACDUAtt (sense); UACGUGUUCAUGCCGUUCAtc (antisense)]. Silencer^®^ Select *GAPDH* siRNA and Negative Control siRNA (Ambion) were used as controls. The efficiency of transfection with lipofectamine RNAiMAX (Invitrogen), repeated once after 3 days, was controlled by co-incubation with Silencer^®^ Cy™3-labeled Negative Control No. 1 siRNA (Ambion). Cells were harvested 5 days post-transfection for qPCR, as indicated above, and western blot analysis. For this purpose, solubilized proteins were separated by SDS-PAGE (4-15%), transferred to polyvinylidene difluoride membranes by Transblot (BioRad) and incubated overnight at 4°C with rabbit antibodies against Foxa1 and Foxa2 (Seven Hills), respectively. Following incubation with horseradish peroxidase-conjugated antibodies, the membranes were developed with enhanced chemiluminescence using Luminata Forte Western (Millipore).

### [^3^H]Thymidine incorporation

[^3^H]Thymidine (1 µCi/ml) was added to TT cells for the last 24 h of siRNA treatment. Radiolabeled cells were extensively washed in ice-cold PBS and dissolved in Soluene-350 (PerkinElmer), after which Ultima Gold LSC Cocktail (PerkinElmer) was added and the radioactivity measured in a PerkinElmer liquid scintillator. The mean±s.e.m. of pooled data from three identical experiments was calculated using a two-sided *t*-test with Bonferroni correction. A corrected *P*-value <0.05 was considered significant.
